# Different duration of parathyroid hormone exposure distinctively regulates primary response genes Nurr1 and RANKL in osteoblasts

**DOI:** 10.1371/journal.pone.0208514

**Published:** 2018-12-21

**Authors:** Hyewon Choi, Clara E. Magyar, Jeanne M. Nervina, Sotirios Tetradis

**Affiliations:** 1 Division of Oral Biology and Medicine, School of Dentistry, University of California at Los Angeles, Los Angeles, California, United States of America; 2 Center for Pathology Research Services, Department of Pathology, University of California at Los Angeles, Los Angeles, California, United States of America; 3 College of Dentistry, New York University, New York, New York, United States of America; 4 Division of Diagnostic and Surgical Sciences, School of Dentistry, University of California at Los Angeles, Los Angeles, California, United States of America; Universite de Nantes, FRANCE

## Abstract

Parathyroid hormone (PTH) exerts dual effects, anabolic or catabolic, on bone when administrated intermittently or continuously, via mechanisms that remain largely unknown. PTH binding to cells induces PTH-responsive genes including primary response genes (PRGs). PRGs are rapidly induced without the need for de novo protein synthesis, thereby playing pivotal roles in directing subsequent molecular responses. In this study, to understand the role of PRGs in mediating osteoblastic cellular responses to PTH, we investigated whether various durations of PTH differentially induce PRGs in primary osteoblasts and MC3T3-E1. Nurr1 and RANKL, PRGs known for their anabolic and catabolic roles in bone metabolism respectively, presented distinctive transient vs. sustained induction kinetics. Corroborating their roles, maximum induction of Nurr1 was sufficiently achieved by brief PTH in as little as 30 minutes and continued beyond that, while maximum induction of RANKL was achieved only by prolonged PTH over 4 hours. Our data suggested distinctive regulatory mechanisms for Nurr1 and RANKL: PKA-mediated chromatin rearrangement for transcriptional regulation of both PRGs and ERK-mediated transcriptional regulation for RANKL but not Nurr1. Lastly, we classified PRGs into two groups based on the induction kinetics: The group that required brief PTH for maximum induction included Nur77, cox-2, and Nurr1, all of which are reported to play roles in bone formation. The other group that required prolonged PTH for maximum induction included IL-6 and RANKL, which play roles in bone resorption. Together, our data suggested the crucial role of PRG groups in mediating differential osteoblastic cellular responses to intermittent vs. continuous PTH. Continued research into the regulatory mechanisms of PKA and ERK for PRGs will help us better understand the molecular mechanisms underlying the dual effects of PTH, thereby optimizing the current therapeutic use of PTH for osteoporosis.

## Introduction

Parathyroid hormone (PTH), an endocrine regulator of calcium homeostasis, exerts paradoxical dual effects, anabolic or catabolic, on bone metabolism depending on whether it is administered intermittently or continuously [1, 2]. Although PTH increases bone turnover, intermittent PTH upregulates osteoblast differentiation and function more than osteoclastogenesis, leading to net bone gain. In contrast, continuous PTH significantly enhances osteoclastogenesis and to a lesser extent osteoblastogenesis leading to net bone loss [3–5]. However, the differential cellular responses that occur during the hours following the administration of intermittent vs. continuous PTH remain largely unknown, hindering a comprehensive understanding of the dual effects of PTH.

Upon binding to osteoblasts, PTH’s primary target cells, PTH rapidly activates PTH-signaling cascades including protein kinase A (PKA), protein kinase C (PKC) as well as MEK (MAPK/ERK kinase)/ ERK (extracellular signal-regulated kinase) pathways to induce PTH-responsive genes including ‘first-responder’ primary response genes (PRGs) [6]. PRGs are genes that are rapidly induced without the need for de novo protein synthesis [7]. They affect subsequent molecular responses by playing versatile roles as transcription factors, enzymes, signaling mediators, and cytokines in various types of cells, such as neuronal cells, cardiac cells, and immune cells [8–11]. Among PRGs, Nurr1 and RANKL are known to play distinctive roles in osteoblasts and bone; Nurr1, a member of the NR4A nuclear orphan receptor family, is involved in osteoblastic differentiation, as shown by its transactivation of osteocalcin and osteopontin and its upregulation during osteoblastic differentiation [9, 10, 12–14]. RANKL, a member of the tumor necrosis factor (TNF) cytokine family, is a well-known master regulator of osteoclastogenesis [15].

In this study, to understand the role of PRGs in mediating osteoblastic cellular responses to intermittent vs. continuous PTH, we investigated the induction kinetics of PRGs for brief vs. prolonged PTH as well as the regulatory mechanisms for PRGs, specifically Nurr1 and RANKL, in primary calvarial osteoblasts (pOBs) and the MC3T3-E1 osteoblastic cell line. Cells were treated with PTH either for a prolonged period (the entire duration of the experiment) or for only brief periods. Distinctive transient vs. sustained induction kinetics for brief vs. prolonged PTH, as well as distinctive regulatory mechanisms of the PTH-signaling mediators PKA vs. MEK/ERK were observed for Nurr1 vs. RANKL. Continued investigation of other PRGs revealed two groups of PTH-induced osteoblastic PRGs. One group included Nur77, COX-2, and Nurr1, all of which required only brief PTH for maximum induction; the other group included IL-6 and RANKL, which required prolonged PTH for maximum induction. Overall, our study demonstrated differential induction of PRGs in response to brief vs. prolonged PTH, which may in turn play a crucial role in differential osteoblastic cellular responses and consequently, in the dual effects of PTH on bone.

## Materials and methods

### Cell culture and reagents

Primary calvarial osteoblasts (pOBs) were isolated from 6–8 day old CD-1 neonatal mice (Charles River laboratories, Inc., Boston, MA) and cultured as previously described [13]. All animals used in our studies were euthanized according to protocol approved by UCLA Institutional Animal Care and Use Committee (ARC No. 98–175–02). pOBs were plated at a concentration of 35,000~40,000 cells/cm^2^ and cultured in DMEM with 10% FBS and 1% antibiotics (100 units/ml penicillin and 50 μg/ml streptomycin) for 10 days to reach confluency. MC3T3-E1 osteoblastic cells were obtained from Riken Cell Bank (Japan) and cultured as previously described [16]. For all experiments, MC3T3-E1 cells were plated at a density of 60,000 cells/cm^2^ and cultured in α-Minimum-EM (Gibco, A10490) with 10% FBS and 1% antibiotics and cultured for 5 days to reach confluency. Medium was changed every two to three days. Prior to the experiments, cells were serum-starved overnight by changing medium to 1% FBS and 1% antibiotics, then treated with 10nM bovine PTH (1–34) (Sigma-Aldrich, St. Luis, MO) and/or 30 μM PKA inhibitor H89 (Sigma-Aldrich, #B1427) or 10μM MEK/ERK inhibitor U0126 (Sigma-Aldrich, #V1121) or 2 μM actin polymerization inhibitor Cytochalasin D (Calbiochem, #250255) or 2 μM Rho kinase (ROCK) inhibitor Y-27632 (Calbiochem, #688000) 15minutes prior to PTH for pre-treatment or 1 or 2 hours after PTH for post-treatment.

### RNA extraction and real-time quantitative PCR (qPCR)

Total RNA from cell culture was collected using Trizol (Invitrogen, Carlsbad, CA), reverse-transcribed, and prepared for qPCR as previously described [13]. qPCR was performed using iQ SYBR Green master mix (BioRad, Hercules, CA) and gene-specific primers (S1 Table) in triplicates for at least 3 independent experiments. Data analysis was performed using the iCycler System and iCycler iQ Optical System software (BioRad, Hercules, CA). Relative gene induction was determined by the 2−ΔΔCtmethod as previously described [17]. Gene expression levels were normalized to GAPDH and shown as the percentage relative to the maximum expression level. Instead of log fold change, this percentage maximum was chosen to avoid large and artificial fluctuations of gene induction in cases the control levels were low.

### Chromatin immunoprecipitation (CHIP) assay

Confluent cells were subjected to ChIP assay using the ChIP assay kit (Upstate, Biotechnology, Charlottesville, VA) following the manufacturer’s protocol with few modifications. Briefly, cells were treated with 1% formaldehyde solution for 10 min at room temperature to crosslink chromatin, then neutralized by 0.125 M Glycine for 3 min at RT, washed twice with cold PBS, and collected in PBS with protein inhibitors. About 1 million MC3T3-E1 cells were lysed in 200 μl of SDS nuclei lysis buffer. About 2–3 million primary cells collected in PBS were lysed in a nuclei lysis buffer (5 mM PIPES, 85mM KCL, 0.5% NP-40) containing protease inhibitors. After incubating on ice for 10 min, cells in lysis buffer were centrifuged to collect the pellets, which were resuspended in 200 μl of SDS lysis buffer. Lysates containing chromatin were sonicated to obtain DNA fragments ranging from 300–800 bp with the average of 500 bp. Chromatin was immunoprecipitated overnight at 4°C using antibodies against acetylated Histone H4 (Upstate). Then the antibody-chromatin complexes were recovered by Protein A DNA beads (Upstate), washed, extracted in elution buffer supplemented with proteinase K, then further reverse-crosslinked, purified with phenol/chloroform extraction, and dissolved in 20 μl of Nuclease-free water to be further analyzed by PCR or qRT-PCR.

### Polymerase chain reaction (PCR) for ChIP assay

5 μl of ChIP assay samples were used for PCR reaction using Taq polymerase (Promega Corporation, Fitchburg, WI). Reaction volume of total 25 μl (1X buffer, 2 mM MgCl, 0.5μl Taq polymerase, 0.5 μM of each primers, 0.2 mM dNTP) was prepared for PCR amplification. PCR cycle was run for 27 cycles for Nurr1, and 30 cycles for RANKL. Primer sequences are shown in S1 Table. Samples were loaded onto 1.8% agarose gel or 8% acrylamide gel. DNA on gels was stained with EtBr solution to be visualized.

### Restriction enzyme accessibility assay (REA)

Restriction enzyme accessibility assays were performed as described previously [18]. Briefly, primary osteoblasts cultured for 10 days were washed twice with cold PBS and scraped in 1.5 ml of cold PBS. Cells were pelleted at 2000 rpm for 5 min at 4°C and supernatants were discarded. Then cell pellets were resuspended in 1 ml of NP-40 lysis buffer (10 mM Tris-HCl pH 7.4, 10 mM NaCl, 3 mM MgCl_2_, 0.5% NP-40, 0.15 mM spermine, 0.5 mM spermidine) and incubated on ice for 5 min. Tubes were centrifuged at 2000 rpm for 10 min at 4°C to isolate nuclei. The nuclei pellets were resuspended in RE digestion buffer (10 mM Tris-HCl pH 7.4, 50 mM NaCl, 10 mM MgCl_2_, 0.2 mM EDTA, 0.2 mM EGTA, 0.15 mM spermine, 0.5 mM spermidine, 1 mM beta-mercaptoethanol), centrifuged at 2000 rpm for 10 min at 4°C. The supernatants were discarded to isolate nuclei pellets. This was resuspended in 50 μl of 1X New England Biolab (NEB) buffer and incubated with limiting amounts of restriction enzyme (100 U) for 15 min at 37°C, followed by genomic DNA isolation using Qiagen DNeasy kit. Purified DNA (10–15 μg) was digested to completion to generate reference cleavage products using the following restriction enzymes: KpnI and PstI for RANKL, and HindIII and XhoI for Nurr1. Samples were analyzed by Southern blot with 32P-labeled gene-specific probes following previously published protocol [18].

### Rhodamin-phalloidin staining

Cells were plated at a concentration of 35,000~40,000 cells/cm^2^ in DMEM in the 12 well plates containing glass coverslips, cultured for 3 days, serum-starved overnight and treated with PTH. For staining, cells were washed once with PBS, fixed for 1min at RT with 4% formaldehyde, permeabilized with 0.5% Triton X-100 and stained with Rhodamin-phalloidin (Thermo Fisher scientific, Waltham, MA) to visualize filamentous actin. Slides were mounted using Vectashield (Vector Laboratories, Burlingame, CA) and analyzed on a Leica Microscope (Leica Manheim, Germany).

### Rho activation assay

Rho activity was determined by the pull-down assay using Rho activation assay kit (Upstate, USA) following manufacturer’s protocol. Subsequent immunoblot analysis using the anti-Rho antibody to visualize the level of active RhoA was performed.

### Statistical analysis

Data were expressed as mean ± standard error of the mean (SEM) from at least 3 independent experiments. To determine the statistical significance, student’s T test with one tail analysis was used for two groups (control and the experimental group) and one-way ANOVA with Student-Newman-Keuls post-hoc test was used for multiple groups. P values less than 0.05 were considered statistically significant.

## Results

### Differential induction kinetics of Nurr1 and RANKL in response to brief vs. prolonged PTH

**Fig 1:**
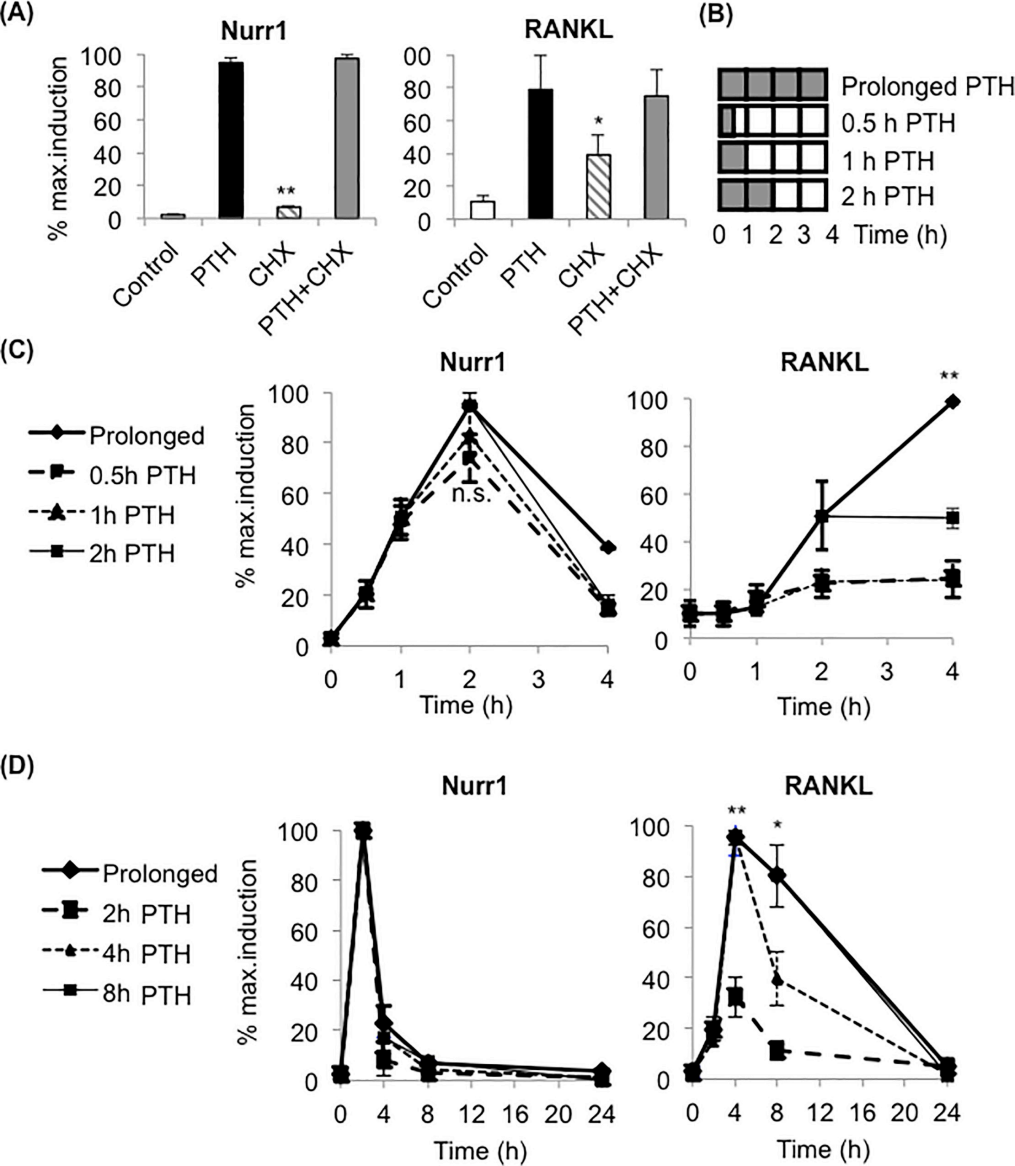
Primary response genes Nurr1 and RANKL were differentially induced by brief vs. prolonged PTH in osteoblasts. **(A) qPCR analysis of Nurr1 (left) and RANKL (right) mRNA expression in pOBs.** Cells were pre-treated with 3μg/ml Cycloheximide (CHX) for 30 minutes and subsequently with PTH for 1 hour (Nurr1) or 2 hours (RANKL). Values were normalized by GAPDH and presented as a percentage of the maximum expression level. Results indicated that Nurr1 and RANKL are PTH-induced primary response genes in pOBs (n = 3, mean±SEM, *p<0.05, **p<0.01). **(B) Schematic diagram showing brief vs. prolonged PTH treatment regimes for Fig 1(C).** Cells were treated with PTH (indicated as gray bars) for various periods of time, washed twice with PBS, and changed into a PTH-free medium (blank bar) for brief PTH or treated with PTH throughout the time-course for prolonged PTH. Cells were collected altogether at the end of time-course. **(C,D) qPCR analysis of Nurr1 (left) and RANKL (right) mRNA expression in pOBs treated with brief or prolonged PTH.** In the 4-hour time course (C), cells were treated with either prolonged PTH for 4 hours or brief PTH for 0.5 to 2 hours. In the 24-hour time course (D), cells were treated with either prolonged PTH for 24 hours or brief PTH for 2 to 8 hours. The Nurr1 expression level peaked at 2 hours, and the maximum expression levels induced by all brief vs. prolonged treatment regimes were not significantly different. The RANKL expression level peaked at 4 hours, and the maximal expression level was achieved after at least 4 hours of PTH. Values were normalized by GAPDH and presented as a percentage of the maximum expression level (n = 5, mean±SEM, *p<0.05, **p<0.01).

We aimed to examine whether brief vs. prolonged PTH differentially induce Nurr1 and RANKL in osteoblasts. First, we tested whether Nurr1 and RANKL were PTH-induced PRGs in primary calvarial osteoblasts (pOBs) and MC3T3 osteoblastic cells. The PTH-induced mRNA expression levels of both Nurr1 and RANKL were not attenuated by the pre-treatment of cycloheximide (CHX), a protein synthesis inhibitor; therefore, Nurr1 and RANKL were confirmed as PTH-induced osteoblastic PRGs (Fig 1A, S1 Fig). Next, we examined the induction kinetics of Nurr1 and RANKL following various durations of PTH treatment. Cells were treated with PTH either for a prolonged period (the entire duration of the experiment) or for only brief periods (Fig 1B). To ensure brief exposure, cells were washed twice with PBS and changed into PTH-free media for the remainder of the experiment. As indicated by the PTH ELISA assay, the relative amount of PTH in the cultured osteoblasts after washes was not statistically different from the baseline (S2 Fig). Within a 4-hour time course, none of the maximum Nurr1 levels, induced by 2 hours of brief or prolonged PTH, were significantly different in pOBs (Fig 1C, left panel) or the MC3T3 cell line (S3 Fig, left panel). However, maximum induction of RANKL required prolonged PTH exposure, as the maximum RANKL levels induced at 4 hours by brief PTH from 0.5 hour to 2 hours were significantly lower than the maximum RANKL level induced by prolonged PTH in pOBs (Fig 1C, right panel) and the MC3T3 cell line (S3 Fig, right panel). When the time course was extended to 24 hours, it was found that RANKL required the prolonged presence of PTH for at least 4 hours (Fig 1D).

### Transcriptional regulation of Nurr1 and RANKL via chromatin remodeling

**Fig 2:**
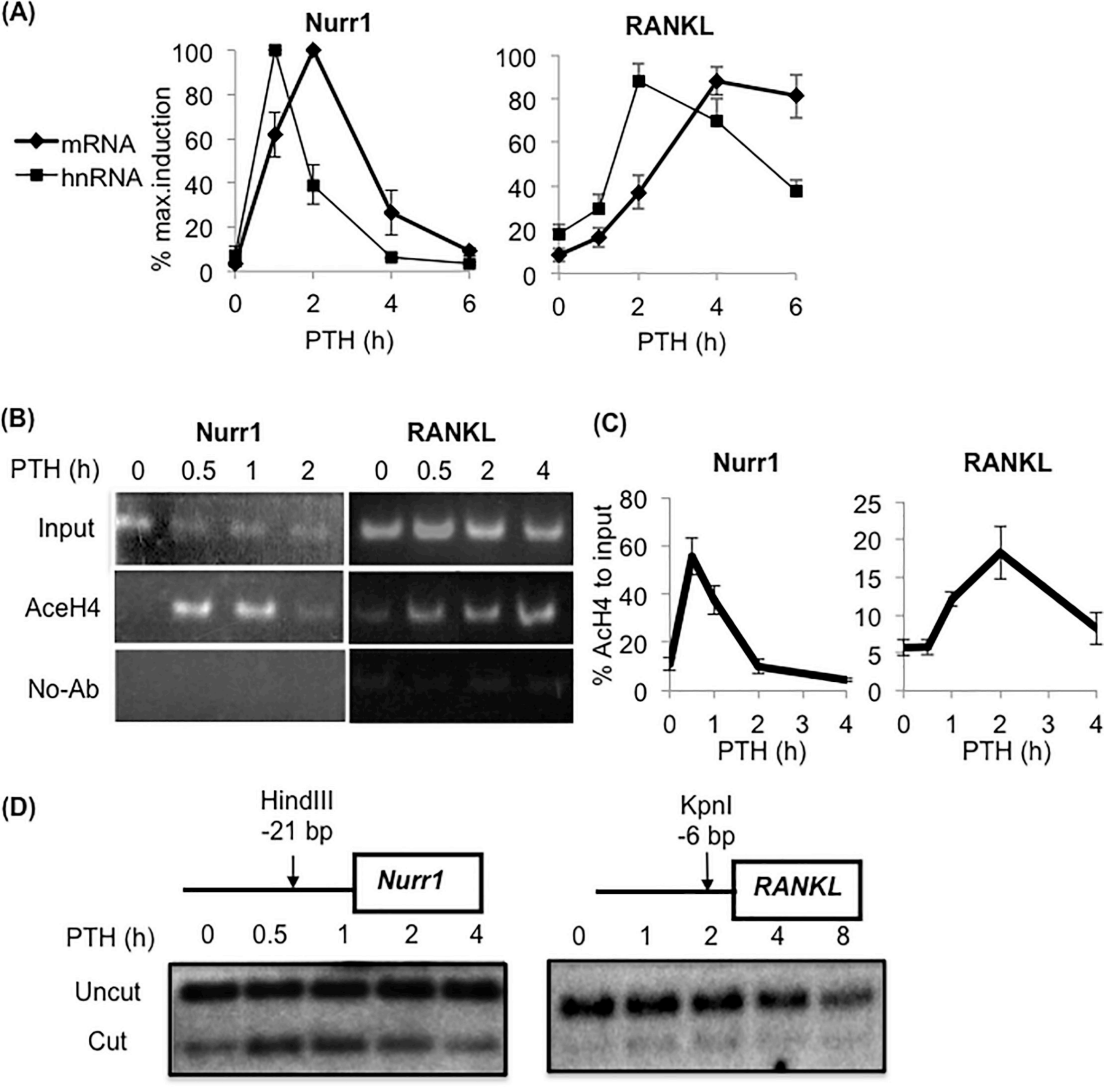
PTH induced histone H4 acetylation and chromatin decondensation near the transcription start sites of Nurr1 and RANKL for transcriptional regulation. **(A) qPCR analysis of PTH-induced Nurr1 (left) and RANKL (right) mRNA and heteronuclear RNA (hnRNA) levels in pOBs.** mRNA and hnRNA levels were determined by qPCR using primer sets amplifying exon-exon and exon-intron junction, respectively. Values were normalized by GAPDH and presented as a percentage of the maximum expression level (n = 5, mean±SEM). **(B,C) PTH-stimulated changes in histone H4 acetylation near transcription start sites of Nurr1 (left panels) and RANKL (right panels).** For the ChIP assay, cross-linked chromatins from pOBs treated with PTH for the indicated number of hours were immunoprecipitated with anti-acetylated Histone H4 antibody or without the antibody. Immunoprecipitated chromatins were reverse-crosslinked and subjected to PCR (B) or qPCR (C) analysis with primers amplifying near the transcription start site of Nurr1 and RANKL (primer sequences indicated in S1 Table). Representative acrylamide gel images of PCR analysis (B) from three independent experiments with similar results are shown. For qPCR, values are shown as a percentage of the input (n = 3, mean±SEM). Note that the kinetics of Histone H4 acetylation and hnRNA levels are similar for both Nurr1 and RANKL. **(D) The restriction endonuclease assay (REA) to monitor PTH-induced nucleosome remodeling near the transcription start site of Nurr1 and RANKL.** The schematic diagram shows the locations of the restriction enzyme HindIII and KpnI recognition sites used for Nurr1 and RANKL, respectively (top panels). For REA, nuclear lysates from POBs treated with PTH for the indicated number of hours were digested with indicated restriction enzymes and subjected to southern blot analysis with a gene-specific 32P-labled probe for -100 bp to +50bp regions of Nurr1 and RANKL. Representative images from three independent experiments with similar results are shown. Note that the kinetics between Histone H4 acetylation, hnRNA level, and PTH-induced nucleosome remodeling are similar for both Nurr1 and RANKL.

To better understand the transcriptional regulatory mechanism of Nurr1 and RANKL in osteoblasts, we compared short-existing unspliced heteronuclear RNA (hnRNA) levels with the mRNA levels of Nurr1 and RANKL (Fig 2A). The hnRNA level was measured by qPCR with primer sets amplifying the exon-intron junctions (S1 Table). For both genes, the kinetics of hnRNA and mRNA were similar, while maximal hnRNA levels were detected earlier than those of mRNA. Together, these findings suggest that transcription rate is the major determinant of Nurr1 and RANKL mRNA expression level. To further understand the regulatory mechanisms of PRG transcription, we examined PTH-induced histone acetylation and nucleosomal rearrangement near the transcription start site with Chromatin immunoprecipitation (ChIP) and Restriction enzyme (REA) assays, respectively. Acetylation of histone H4 has been reported to be a marker of active transcription, and strongly associated with open chromatin structures [19, 20]. ChIP indicated that PTH induced transient acetylation of histone H4 near the transcription start site of both genes. The acetylation level peaked between 0.5 and 1 hour for Nurr1 and between 2 and 4 hours for RANKL in both PCR (Fig 2B) and qPCR analysis (Fig 2C). In REA, we observed that restriction enzyme cleavage efficiency peaked between 0.5 and 1 hour for Nurr1 and at 2 hours for RANKL, and returned to the basal level within 4 hours for Nurr1 and 8 hours for RANKL (Fig 2D). These observations indicated that PTH induced transient chromatin remodeling, allowing increased access to genomic regions near the transcription start site of Nurr1 and RANKL. For both genes, there were strong commonalities in time points among hnRNA, histone H4 acetylation and the nucleosomal rearrangement kinetics of each gene. Taken altogether, these data suggest that PTH-induced Nurr1 and RANKL expression is regulated at the transcription level, at least in part, through local chromatin remodeling near transcription start sites.

### PKA-mediated regulation of local histone acetylation and the expression of Nurr1 and RANKL

**Fig 3:**
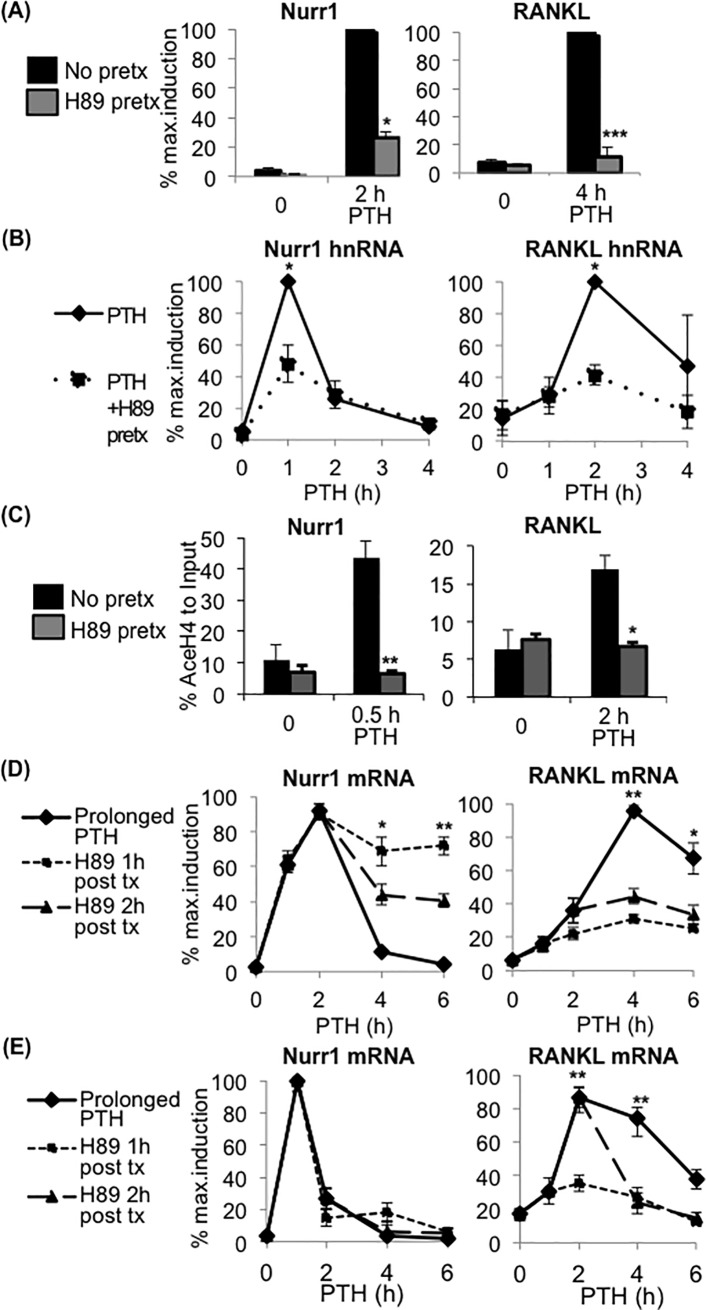
Inhibition of PKA affected transcription and histone H4 acetylation near the transcription start sites of both Nurr1 and RANKL. **(A,B) qPCR analysis of the mRNA level (A) and hnRNA level (B) of Nurr1 (left) and RANKL (right) in POBs pre-treated with PKA inhibitor H89 15 minutes prior to PTH treatment.** hnRNA were determined by qPCR using primer sets amplifying exon-intron junction. Note that the maximum mRNA and hnRNA levels of both Nurr1 and RANKL were significantly down-regulated by H89 pre-treatment. Values were normalized by GAPDH and presented as a percentage of the maximum expression level (n = 3, mean±SEM, *p<0.05, **p<0.01). **(C) PTH-stimulated changes in histone H4 acetylation near transcription start sites of Nurr1 (left panels) and RANKL (right panels).** For the ChIP assay, cross-linked chromatins from pOBs treated with PTH for indicated number of hours were immunoprecipitated with the anti-acetylated Histone H4 antibody or without the antibody. Immunoprecipitated chromatins were reverse-cross-linked and subjected to qPCR analysis with primers amplifying near the transcription start sites of Nurr1 and RANKL (primer sequences indicated in S1 Table). Values were shown as a percentage of the input. Note that the Histone H4 acetylation level was significantly down-regulated by H89 pre-treatment for both Nurr1 and RANKL (n = 3, mean±SEM, *p<0.05, **p<0.01). **(D,E) qPCR analysis of mRNA level (D) and hnRNA level (E) of Nurr1 (left) and RANKL (right) in POBs received PKA inhibitor H89 post-treatment 1 or 2 hours after PTH treatment.** Note that the maximum mRNA and hnRNA levels of Nurr1 were not affected by H89 post-treatment. Values were normalized by GAPDH and presented as a percentage of the maximum expression level (n = 5, mean±SEM, *p<0.05, **p<0.01).

PKA is a major regulator of PTH-response gene expression in several PTH-responsive cells [21, 22]; therefore we examined whether PKA mediated Nurr1 and RANKL expression in osteoblasts by pre-treating cells with 30 μM H89, a widely-used PKA chemical inhibitor [23–25], prior to PTH treatment. H89 pre-treatment significantly attenuated PTH-induced Nurr1 and RANKL expression in POBs (Fig 3A) and also in the MC3T3-E1 cell line (S4A Fig), suggesting that PKA mediated PTH-induced Nurr1 and RANKL expression in osteoblasts. To further investigate the regulatory mechanism of PKA, we examined whether H89 pre-treatment affected hnRNA levels and histone H4 acetylation near the transcription start sites of Nurr1 and RANKL. As expected, H89 pre-treatment significantly reduced both Nurr1 and RANKL hnRNA levels (Fig 3B). Similarly, Histone H4 acetylation levels for both Nurr1 and RANKL were significantly inhibited by H89 pre-treatment (Fig 3C**)**. Together these data suggest that PKA regulates the transcription of both Nurr1 and RANKL at least in part by local histone acetylation associated with chromatin remodeling.

We also tested the time-dependent requirements of PKA for Nurr1 and RANKL expression by H89 post-treatment, which allowed PTH to initiate PKA signaling. For Nurr1, H89 post-treatment did not affect the maximum mRNA levels and, interestingly, upheld mRNA levels even after peaks in pOBs (Fig 3D, left panel) and the MC3T3 cell line (S4B Fig, left panel). Yet H89 post-treatment did not affect Nurr1 hnRNA (Fig 3E, left panel), indicating that H89 post-treatment enhanced Nurr1 mRNA levels via a post-transcriptional regulatory mechanism. For RANKL, H89 post-treatment significantly attenuated the maximum mRNA and hnRNA levels in pOBs (Fig 3D and 3E, right panel) and the MC3T3 cell line (S4B Fig, right panel). Altogether, these data suggest that initial activation of PKA signaling was sufficient to induce maximum Nurr1 transcription, while continuous activation of PKA signaling was required for maximum RANKL transcription.

### MEK/ERK-mediated regulation of RANKL but not Nurr1

**Fig 4:**
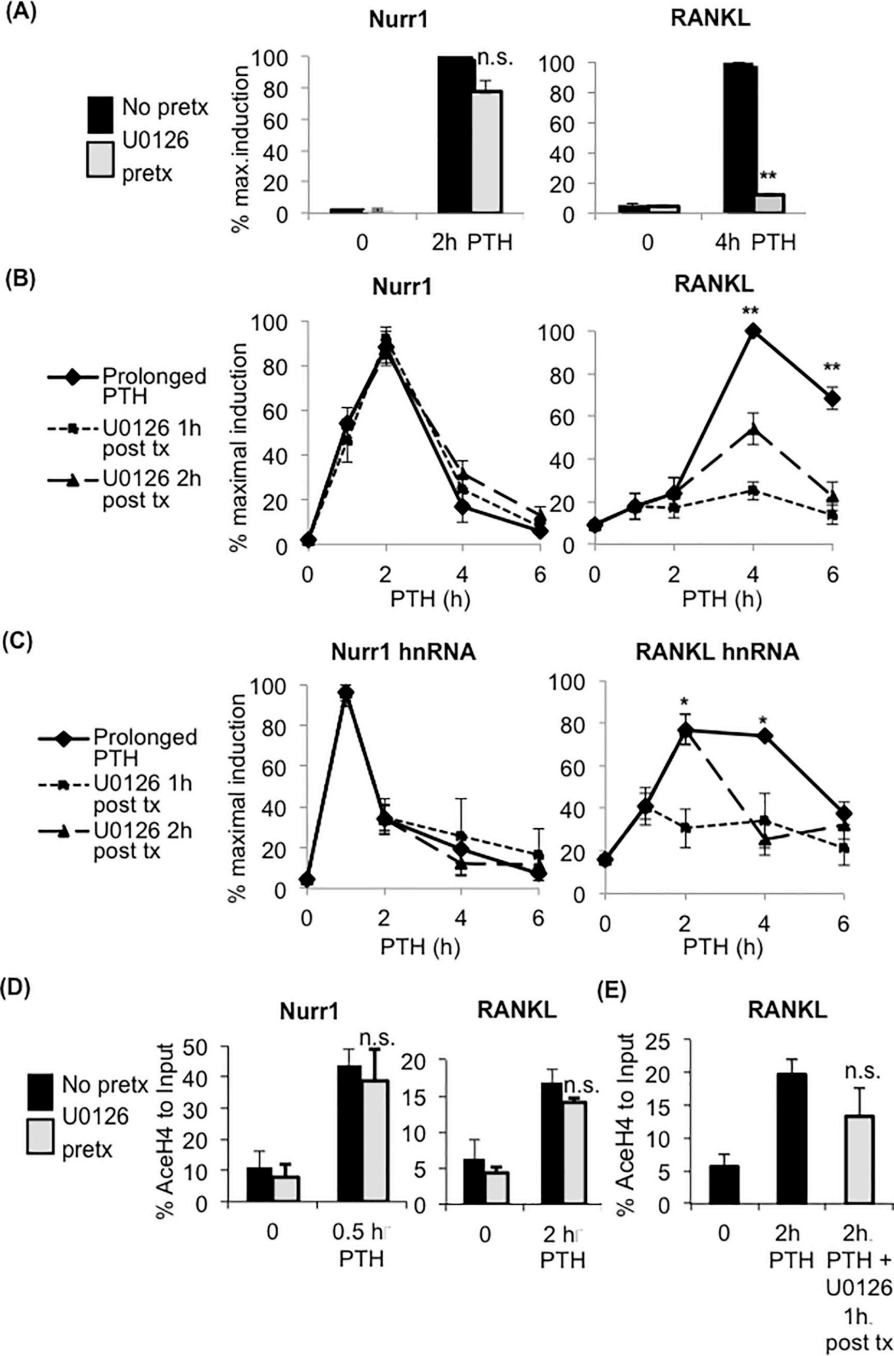
Inhibition of ERK affected RANKL transcription, but not Nurr1. **(A-C) qPCR analysis of mRNA level (A,B) and hnRNA level (B) of Nurr1 (left) and RANKL (right) in pOBs received MEK inhibitor U0126 pre-treatment** (A) 15 minutes prior to PTH or post-treatment (B,C) 1 or 2 hours after PTH. The level of hnRNA was determined by qPCR using primer sets amplifying exon-intron junction. Note that the maximum mRNA and hnRNA levels of only RANKL, but not Nurr1, were significantly down-regulated by U0126 pre-and post-treatment. Values were normalized by GAPDH and presented as a percentage of the maximum expression level (n = 5, mean±SEM, *p<0.05, **p<0.01). **(D,E) No significant effects of MEK inhibitor U0126 treatment on PTH-induced histone H4 acetylation level near transcription start sites of Nurr1 and RANKL.** Cross-linked chromatins from pOBs, treated with PTH for the indicated number of hours with either U0126 pre-treatment (A) or post-treatment (B,C) were immunoprecipitated with the anti-acetylated Histone H4 antibody or without the antibody. Immunoprecipitated chromatins were reverse-cross-linked and subjected to qPCR analysis with primers amplifying near the transcription start sites of Nurr1 and RANKL (primer sequences indicated in S1 Table). Values are shown as a percentage of the input (n = 5, mean±SEM, not significant (n.s.)).

Another crucial signaling cascade that mediates the effects of PTH is the MEK/ERK pathway. MEK/ERK is implicated in osteoblast proliferation and differentiation, as well as regulation of osteoblastic genes such as RANKL [26–28]. We investigated whether MEK/ERK differentially mediates PTH-induced Nurr1 and RANKL expression in osteoblasts by pre-treating cells with 10μM U0126, a widely used selective inhibitor of MEK/ERK [29–31], prior to PTH treatment. U0126 pre-treatment did not affect Nurr1 mRNA expression, but significantly attenuated RANKL mRNA expression in pOBs (Fig 4A) and in MC3T3 cell line (S4A Fig). Similarly, U0126 post-treatment had no effect on Nurr1 mRNA expression, but significantly inhibited RANKL mRNA expression in pOBs (Fig 4B) and the MC3T3 cell line (S4B Fig). To further explore how MEK/ERK signaling differentially regulates Nurr1 and RANKL transcription, we examined the effects of U0126 post-treatment on hnRNA levels and histone H4 acetylation. Consistent with the regulation of mRNA levels, U0126 post-treatment did not affect Nurr1, but significantly reduced RANKL hnRNA levels (Fig 4C). Also, neither U0126 pre- nor post-treatment affected histone H4 acetylation levels near the transcription start sites of Nurr1 or RANKL (Fig 4D and 4E). Together these data suggest that MEK/ERK regulates selective PRGs, and that continuous activation of MEK/ERK is required for the complete activation of RANKL transcription.

### Rho/ROCK-mediated regulation of RANKL but not Nurr1

**Fig 5:**
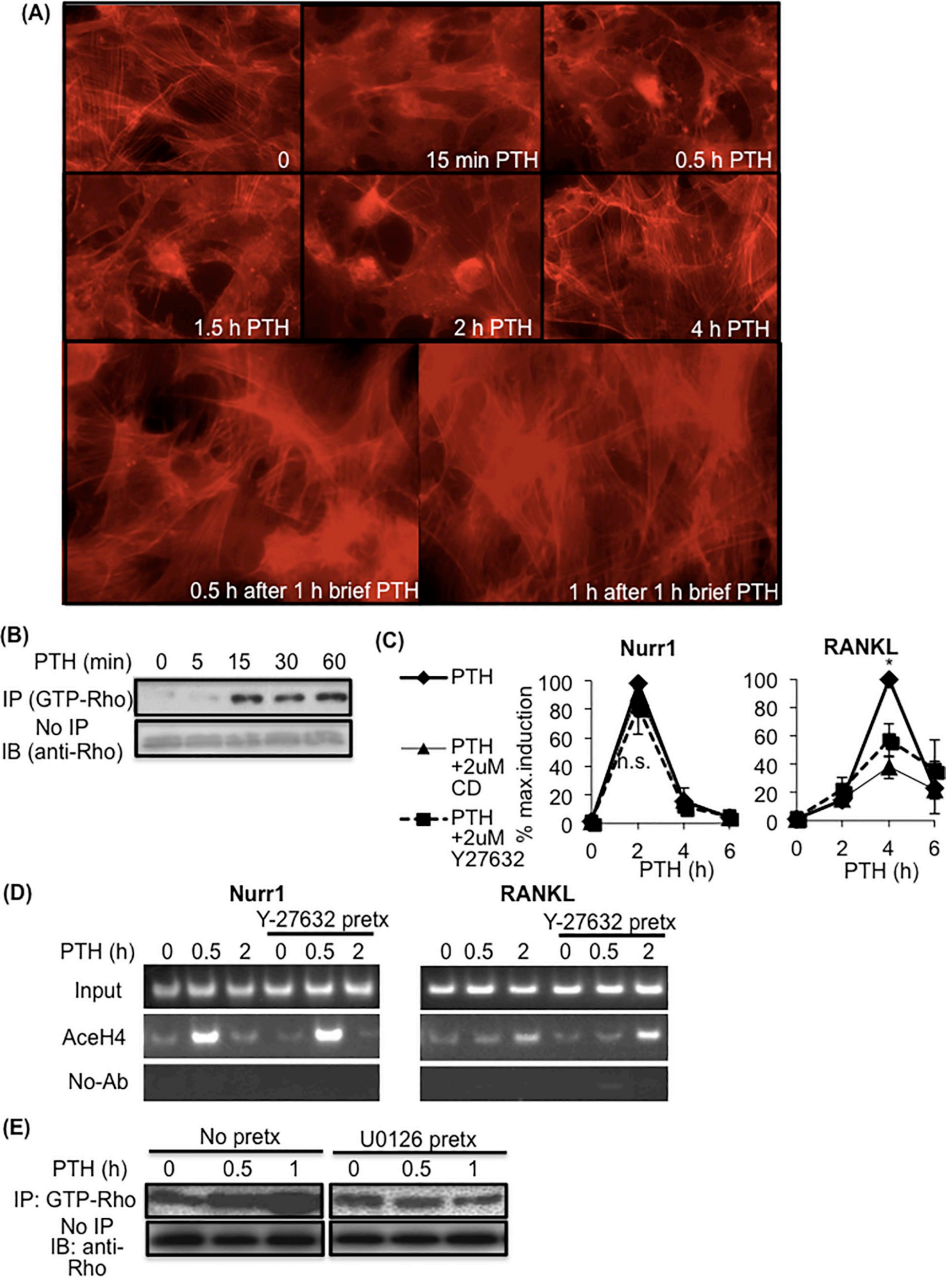
PTH-induced RANKL expression level was down-regulated by inhibition of actin polymerization inhibitor Cytochalasin D and ROCK inhibitor Y-27632 treatment. **(A) PTH-induced changes in filamentous actin (f-actin) cytoskeleton arrangement in pOBs.** For visualization of f-actin, cells were treated with either prolonged PTH (PTH) or brief PTH, stained with rhodamin-phalloidin and fixed with 4% formaldehyde to be examined under fluorescence microscopy. For brief PTH, cells were treated with PTH for one hour, changed into a PTH-free medium, and prepared for staining after 30 minutes (bottom left image) or one hour (bottom right image). **(B) PTH-induced changes in the amount of active Rho in pOBs.** Cells treated with PTH for the indicated period of time were prepared for Rho activation assay pulling-down active Rho, which was subsequently detected by immunoblot analysis using the anti-Rho antibody. Note that activation of Rho was rapidly induced by 15 minutes of PTH treatment. A representative immunoblot image from three independent experiments with similar results is shown. **(C) qPCR analysis of Nurr1 (left) and RANKL (right) mRNA level in pOBs treated with 2** μ**M actin polymerization inhibitor Cytochalasin D (CD) or 2** μ**M ROCK inhibitor Y-27632 15 minutes prior to PTH treatment for indicated hours.** Note that the maximum mRNA level of only RANKL, not Nurr1, was significantly down-regulated by CD as well as Y-27632 pre-treatment. Values were normalized by GAPDH and presented as a percentage of the maximum expression level (n = 5, mean±SEM, *p<0.05, **p<0.01). **(D) The effect of ROCK inhibitor Y-27632 treatment on PTH-induced histone H4 acetylation near the transcription start sites of Nurr1 (left panels) and RANKL (right panels).** pOBs pre-treated with 2 μM ROCK inhibitor Y-27632 for 15 minutes and treated with 10 nM PTH for indicated hours. Cross-linked chromatins from POBs, treated with PTH for indicated hours, were immunoprecipitated with anti-acetylated Histone H4 antibody or without the antibody. Immunoprecipitated chromatins were reverse-cross-linked and subjected to PCR analysis using primers amplifying near the transcription start site of Nurr1 and RANKL (primer sequences indicated in S1 Table). PCR products were separated on 8% acrylamide gel. A representative image from three independent experiments with similar results is shown. Note that, for Nurr1 as well as RANKL, no significant changes in Histone H4 acetylation near the transcription start sites were observed with Y27632 pre-treatment. **(E) The effect of MEK inhibitor U0126 treatment on PTH-induced Rho activation.** pOBs, pre-treated with 10 μM MEK inhibitor U0126 15 minutes prior to PTH, were prepared for Rho activation assay and subsequent immunoblot analysis with anti-Rho. No significant increase in PTH-induced Rho activation was noted in cells pre-treated with U0126. Representative immunoblot images from three independent experiments with similar results are shown.

Our observation of the selective inhibitory effect of U0126 on the transcription of RANKL, but not Nurr1, led us to further investigate the regulatory mechanisms of MEK/ERK in osteoblasts. In other PTH-responsive cells, such as kidney cells and immune cells, MEK/ERK interacted with multiple signaling mediators, including ROCK, a main signaling mediator of Rho-GTPase signaling pathway, for gene regulation [26, 32–35]. Prior to investigating whether Rho-GTPase signaling pathway regulates PRGs, we examined whether brief vs. prolonged PTH differentially induce the rearrangement of actin cytoskeleton, which is regulated mainly by Rho-GTPase signaling pathway in osteoblastic cells [36–39]. pOBs were treated with either prolonged PTH or brief PTH (one hour of PTH followed by a PTH-free medium) and stained with rhodamin-phalloidin for visualization of filamentous actin (f-actin) (Fig 5A). Notably, we observed distinctive kinetics of f-actin rearrangement in response to brief vs. prolonged PTH. After pOBs were treated with prolonged PTH, prominent nuclear f-actin was observed in most of them from 0.5 to 2 hours. Cytoplasmic f-actin fibrous structures were dissociated as early as 15 minutes and had largely returned to their basal structures after 4 hours. By contrast, after pOBs were treated with brief PTH, nuclear f-actin in most of them was less defined than it had been in cells treated with prolonged PTH. After only 2 hours of brief PTH (one hour of PTH followed by a PTH-free medium for one hour), the cytoplasmic f-actin fibrous structures had largely returned to their basal structures. These distinctive kinetics of f-actin rearrangement to brief vs. prolonged PTH were similar to those of PTH-induced PRGs, as both were affected by the duration of PTH.

To verify the involvement of actin cytoskeleton in regulating PRGs, we examined whether chemical inhibition of actin polymerization or Rho-Associated Kinase (ROCK), the main effector of Rho-GTPase signaling pathway, affects PTH-induced Nurr1 and RANKL expression levels in pOBs. Pre-treatment with ROCK inhibitor Y27632 and actin polymerization inhibitor Cytochalasin D (Cyto-D) was chosen over post-treatment, given that both Rho activation, as well as actin cytoskeleton rearrangement, had been observed as early as 15 minutes after PTH treatment (Fig 5A and 5B). Notably, both Cyto-D and Y27632 pretreatment (30 minutes prior to PTH) significantly affected the PTH-induced RANKL expression level (Fig 5C, right panel), while neither Cyto-D nor Y27632 affected the PTH-induced Nurr1 expression level (Fig 5C, left panel). Y27632 pre-treatment did not significantly affect PTH-induced histone H4 acetylation, neither near the transcription start site of Nurr1 nor that of RANKL (Fig 5D), suggesting minimal involvement of ROCK in Histone H4 acetylation regulated by PKA. Meanwhile, MEK/ERK inhibition with U0126 pretreatment attenuated the PTH-induced Rho activation levels at 30 and 60 minutes of PTH treatment, suggesting crosstalk between the MEK/ERK and Rho-ROCK signaling pathways (Fig 5E). Altogether, these data highly suggest that the regulatory network of the MEK/ERK and Rho-ROCK signaling pathways selectively mediates PTH-induced mRNA expression of RANKL, but not Nurr1, in osteoblasts.

### Differential induction kinetics of Nurr1, Nur77, COX-2 vs. RANKL and IL-6 to Brief vs. Prolonged PTH

**Fig 6:**
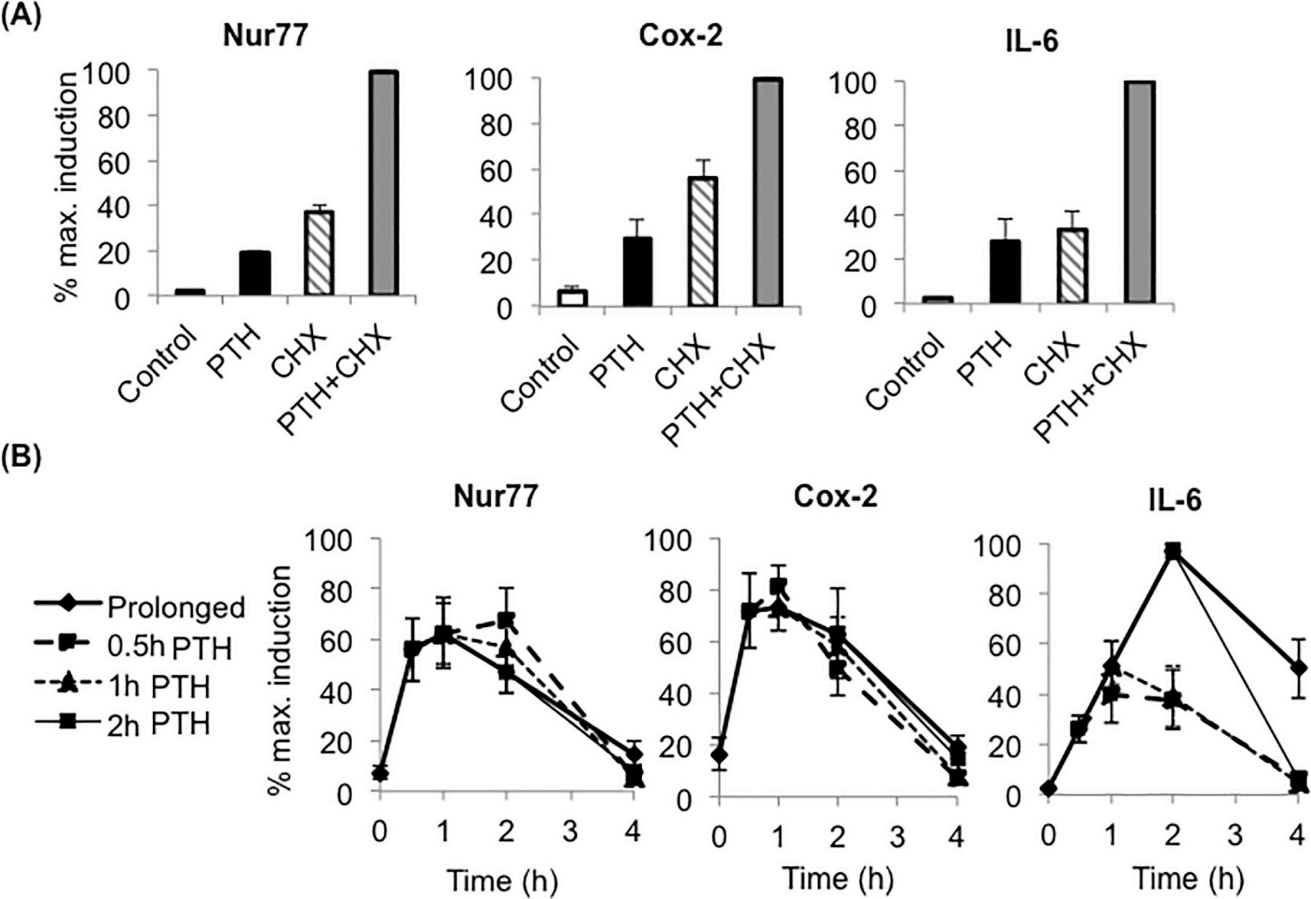
Primary response gene Nur77, cox-2 and IL-6 were differentially induced by brief vs. prolonged PTH, similarly to Nurr1 or RANKL. **(A) qPCR analysis of Nur77, cox-2 and IL-6 mRNA expression in pOBs. Cells were pre-treated with 3**μ**g/ml Cycloheximide for 30 minutes and subsequently with PTH for 2 hours.** Results indicate that all examined genes were PTH-induced primary response genes in pOBs. Values were normalized by GAPDH and presented as a percentage of the maximum expression level (n = 5, mean±SEM). **(B) qPCR analysis of Nur77, cox-2 and IL-6 mRNA expression in pOBs treated with brief or prolonged PTH for 4 hours.** For brief PTH, cells were treated with PTH for 0.5 hour to 2 hours over a 4-hour time course or for 2 to 8 hours over a 24-hour time course, then prepared for qPCR. For prolonged PTH, cells were treated with PTH for 2 hour, washed twice with PBS, and changed into a PTH-containing medium. Note that Nur77 and COX-2 maximal induction was sufficiently achieved by all treatment regimes, while IL-6 maximal induction required at least 2 hours of PTH treatment. Values were normalized by GAPDH and are presented as a percentage of the maximum expression level (n = 5, mean±SEM).

Finally, to expand our observation beyond Nurr1 and RANKL, we examined how other PRGs were differentially induced by brief vs. prolonged PTH in osteoblasts. Nur77, cyclooxygenase-2 (COX-2), and interleukin 6 (IL-6) were confirmed to be PTH-induced PRGs in pOBs, as PTH induced their mRNA expression in both the absence and the presence of protein synthesis inhibitor cyclohexamide (Fig 6A). Similar to Nurr1, maximum induction of Nur77 and COX-2 was achieved in all treatment modes, including brief PTH, in as little as 30 minutes (Fig 6B). Meanwhile, IL-6 required continuous PTH exposure for at least 2 hours for maximum induction (Fig 6B), similar to RANKL. Together, these data suggest the presence of two PRG groups in osteoblasts; one group including Nurr1, Nur77, and COX-2 that required brief PTH for maximum induction, and the other group including RANKL and IL-6 that required prolonged PTH exposure for maximum induction.

## Discussion

Current research on the dual effects of intermittent vs. continuous PTH has demonstrated differential biological responses following multiple rounds of administrations over days, while an initial divergence in molecular responses to each administration has been presumed rather than directly shown. In this study, however, the differential induction kinetics of PRGs for brief vs. prolonged PTH were investigated over shorter periods less than 24 h, indicating two groups of PRGs with distinctive functions in osteoblast biology. Nurr1, Nur77, and COX-2, which required brief PTH as little as 0.5 h for maximum induction, are implicated in anabolic responses by playing positive roles in the proliferation, differentiation, and anti-apoptosis of osteoblasts [40–42]. Meanwhile, IL-6 and RANKL, which required prolonged PTH over 4 h for maximum and sustained induction, play crucial roles in catabolic responses as osteoclastogenesis-stimulating cytokines [43–46]. Our grouping of PRGs is consistent with studies of other cell types, which have likewise reported two groups of PRGs with distinctive induction kinetics [7, 18, 42, 47]. Notably, in macrophages and unstimulated T-cells, the differential inductions of early vs. delayed PRG groups were related to distinctive cellular responses, immediate vs. delayed immune responses [18, 47]. Likewise, it is feasible that the differential induction of PRG groups in osteoblasts contributes to the osteoblasts’ anabolic vs. catabolic responses to intermittent vs. continuous PTH. Yet the role of PRGs in PTH dual effects should be further delineated in the systemic context, as PRGs—including COX-2, which is implicated in both support of osteoblast differentiation and function [42, 48] and osteoclastogenesis [49, 50]—may play multifaceted roles in osteoblasts, osteoclasts and other bone cells, in turn affecting bone metabolism.

**Fig 7:**
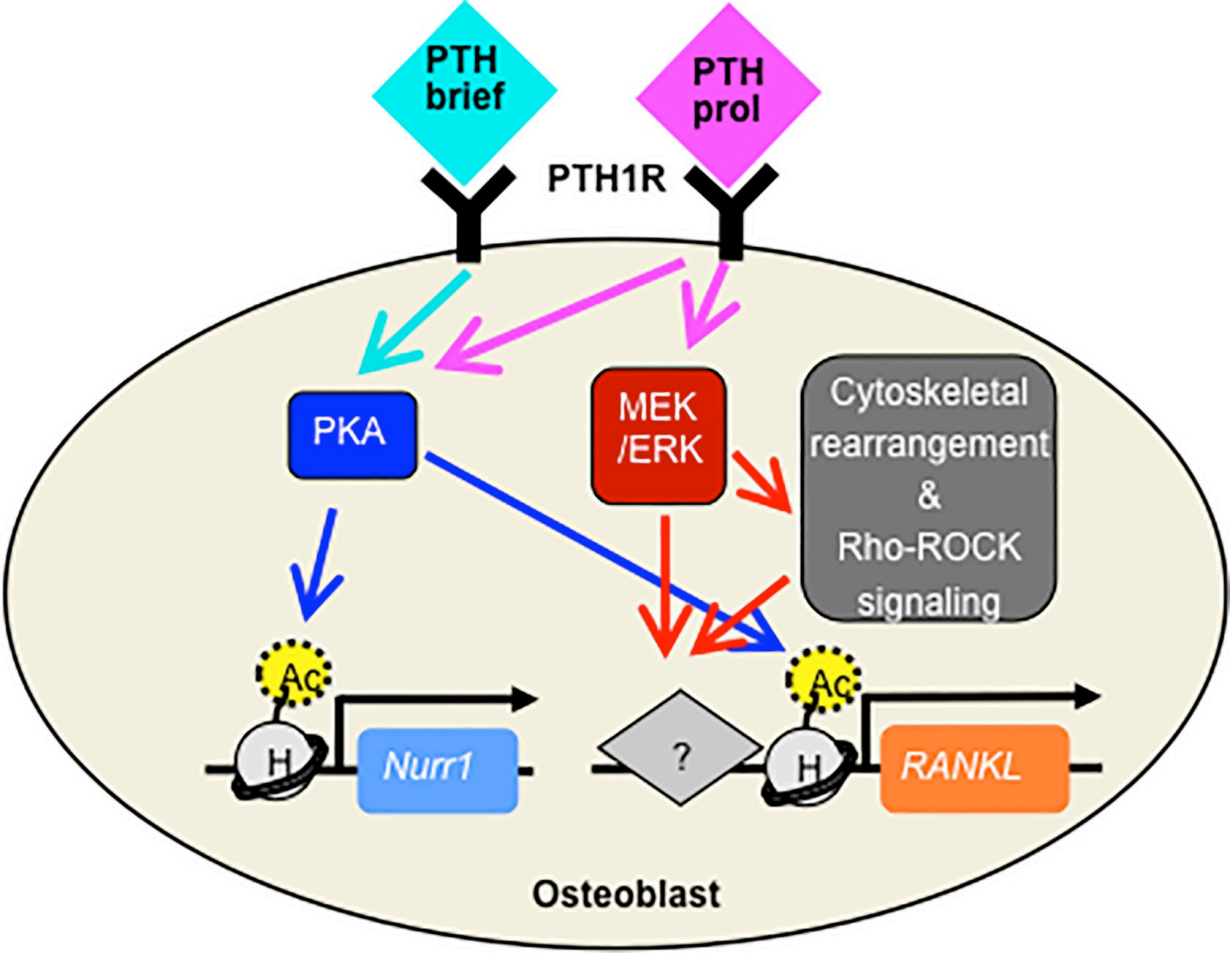
A schematic figure illustrating differential regulatory mechanisms of brief vs. prolonged PTH for Nurr1 and RANKL induction in osteoblasts.

Distinctive regulatory mechanisms of brief vs. prolonged PTH activation of signaling pathways can account for the differential induction of PRG groups. In this study, we identified differential regulation of PKA and MEK/ERK for two representative PRGs, Nurr1 and RANKL. PKA regulated both PRGs at the transcriptional level via chromatin rearrangement, while MEK/ERK selectively regulated RANKL, but not Nurr1, likely via its interaction with the Rho/ROCK signaling pathway and actin cytoskeleton rearrangement, both of which also regulated RANKL, but not Nurr1 (Fig 7). MEK/ERK has been proposed as a crucial regulator of cell fate choices based on its differential activation kinetics to transient vs. sustained stimuli in fibroblasts, neural and carcinoma cells [51–53] and to intermittent vs. continuous PTH in PTH-responsive kidney cells [33, 54]. Moreover, other studies have reported that both MEK/ERK and Rho/ROCK regulate IL-6, a PRG grouped with RANKL in our study, in osteoblastic cells [24, 55, 56]. Together, these data underline the need for investigation into the role of MEK/ERK and other PTH signaling mediators in regulating osteoblastic PRGs and osteoblastic cellular responses to intermittent vs. continuous PTH.

In summary, our study indicates that two PRG groups in osteoblasts were differentially induced by brief vs. prolonged PTH, emphasizing the significance of PRGs in regulating distinctive osteoblastic cellular responses. The PKA, MEK, and Rho/ROCK signaling pathways were examined to demonstrate the distinctive regulations for Nurr1 and RANKL, which play anabolic and catabolic roles in osteoblast biology, respectively. Further investigation of the differential regulatory mechanisms of PRGs by brief vs. prolonged PTH, as well as intermittent vs. continuous PTH will improve understanding of the molecular mechanisms underlying the regulation of osteoblastic gene expression by different regimens of PTH, and might help improve anabolic treatments for osteoporosis.

## Supporting information

S1 Table Primer sequences for PCR and qPCR assay. (TIFF)

S1 Fig Nurr1 and RANKL are PTH-induced PRGs in MC3T3-E1. qPCR analysis of Nurr1 (left) and RANKL (right) mRNA expression in MC3T3-E1 cells. Cells were pretreated with 3μg/ml cycloheximide for 0.5 hour and treated with 10 nM PTH treatment for 2 hours. Results indicated that Nurr1 and RANKL are PTH-induced primary response genes in MC3T3-E1 cells (n = 5, mean±SEM, *p<0.05, **p<0.01).
(TIFF)

S2 Fig PTH ELISA assay to confirm the clearance of PTH from medium after two times of PBS washes. pOBs were treated with PTH for 1 hour followed by indicated number of PBS washes and subjected to PTH ELISA assay. Assay was repeated three times to show.
(TIFF)

S3 Fig Differential induction patterns of Nurr1 and RANKL by brief vs. prolonged PTH in MC3T3-E1. (A,B) qPCR analysis of Nurr1 (left) and RANKL (right) mRNA (A) and hnRNA level (B) in MC3T3-E1 treated with brief or prolonged PTH. MC3T3-E1 cells were treated with 10 nM PTH for 1 hour in 6-hour time course, washed twice with PBS, either changed into PTH-free medium for brief PTH treatment or PTH-containing medium for prolonged PTH, then prepared for qPCR (n = 3, mean±SEM, **p<0.01).
(TIFF)

S4 Fig Differential regulation of PKA and ERK for Nurr1 and RANKL mRNA expression in MC3T3-E1 cells. (A,B) qPCR analysis of PTH-induced Nurr1 (left) and RANKL (right) mRNA level in MC3T3-E1 cells pre- or post-treated with 30 μM PKA inhibitor H89 or 10μM MEK/ERK inhibitor U0126. Pre-treatment (A) was done 15 minutes prior to PTH, and post-treatment (B) was done 1 hour after PTH treatment for indicated hours (n = 3, mean±SEM, *p<0.05,**p<0.01).
(TIFF)

## References

[1] Potts JT. Parathyroid hormone: past and present. Journal of Endocrinology. 2005;187(3):311–25. 10.1677/joe.1.06057 16423810

[2] Silva BC, Bilezikian JP. Parathyroid hormone: anabolic and catabolic actions on the skeleton. Current Opinion in Pharmacology. 2015;22:41–50. 10.1016/j.coph.2015.03.005. 25854704 PMC5407089

[3] Robert LJ, Charles AOB, Ali AA, Paula KR, Robert SW, Stavros CM. Intermittent PTH stimulates periosteal bone formation by actions on post-mitotic preosteoblasts. Bone. 2009;44(2):275–86. S8756-3282(08)00817-X. 10.1016/j.bone.2008.10.037 19010455 PMC2655212

[4] Lotinun S, Sibonga J, Turner R. Differential effects of intermittent and continuous administration of parathyroid hormone on bone histomorphometry and gene expression. Endocr. 2002;17(1):29–36. 10.1385/ENDO:17:1:29 12014700

[5] Ma YL, Cain RL, Halladay DL, Yang X, Zeng Q, Miles RR, et al Catabolic Effects of Continuous Human PTH (1–38) in Vivo Is Associated with Sustained Stimulation of RANKL and Inhibition of Osteoprotegerin and Gene-Associated Bone Formation. Endocrinology. 2001;142(9):4047–54. 10.1210/endo.142.9.8356 11517184

[6] Cheloha RW, Gellman SH, Vilardaga J-P, Gardella TJ. PTH receptor-1 signalling[mdash]mechanistic insights and therapeutic prospects. Nat Rev Endocrinol. 2015;11(12):712–24. 10.1038/nrendo.2015.139 26303600 PMC4651712

[7] Fowler T, Sen R, Roy Ananda L. Regulation of Primary Response Genes. Molecular Cell. 2011;44(3):348–60. 10.1016/j.molcel.2011.09.014. 22055182 PMC3212756

[8] Onan D, Allan EH, Quinn JMW, Gooi JH, Pompolo S, Sims NA, et al The Chemokine Cxcl1 Is a Novel Target Gene of Parathyroid Hormone (PTH)/PTH-Related Protein in Committed Osteoblasts. Endocrinology. 2009;150(5):2244–53. 10.1210/en.2008-1597 19147675

[9] Meir T, Durlacher K, Pan Z, Amir G, Richards WG, Silver J, et al Parathyroid hormone activates the orphan nuclear receptor Nurr1 to induce FGF23 transcription. Kidney Int. 2014;86(6):1106–15. 10.1038/ki.2014.215 24940803

[10] Tetradis S, Bezouglaia O, Tsingotjidou A. Parathyroid Hormone Induces Expression of the Nuclear Orphan Receptor Nurr1 in Bone Cells. Endocrinology. 2001;142(2):663–70. 10.1210/endo.142.2.7926 11159837

[11] Huang JC, Sakata T, Pfleger LL, Bencsik M, Halloran BP, Bikle DD, et al PTH Differentially Regulates Expression of RANKL and OPG. Journal of Bone and Mineral Research. 2004;19(2):235–44. 10.1359/JBMR.0301226 . 14969393

[12] Lee MK, Choi H, Gil M, Nikodem VM. Regulation of osteoblast differentiation by Nurr1 in MC3T3-E1 cell line and mouse calvarial osteoblasts. Journal of Cellular Biochemistry. 2006;99(3):986–94. 10.1002/jcb.20990 16741951

[13] Pirih FQ, Tang A, Ozkurt IC, Nervina JM, Tetradis S. Nuclear orphan receptor Nurr1 directly transactivates the osteocalcin gene in osteoblasts. J Biol Chem. 2004:53167–74. 10.1074/jbc.M405677200 15485875

[14] Lammi J, Huppunen J, Aarnisalo P. Regulation of the Osteopontin Gene by the Orphan Nuclear Receptor NURR1 in Osteoblasts. Mol Endocrinol. 2004;18(6):1546–57. 10.1210/me.2003-0247 14988426

[15] Khosla S. Minireview: The OPG/RANKL/RANK System. Endocrinology. 2001;142(12):5050–5. 10.1210/endo.142.12.8536 11713196

[16] Tseng W, Lu J, Bishop GA, Watson AD, Sage AP, Demer L, et al Regulation of interleukin-6 expression in osteoblasts by oxidized phospholipids. Journal of Lipid Research. 2010;51(5):1010–6. 10.1194/jlr.M001099 PMC2853427. 19965598 PMC2853427

[17] Livak KJ, Schmittgen TD. Analysis of Relative Gene Expression Data Using Real-Time Quantitative PCR and the 2−ΔΔCT Method. Methods. 2001;25(4):402–8. 10.1006/meth.2001.1262. 11846609

[18] Ramirez-Carrozzi VR, Nazarian AA, Li CC, Gore SL, Sridharan R, Imbalzano AN, et al Selective and antagonistic functions of SWI/SNF and Mi-2beta nucleosome remodeling complexes during an inflammatory response. Genes Dev. 2006;20(3):282–96. 10.1101/gad.1383206 16452502 PMC1361700

[19] Kininis M, Chen BS, Diehl AG, Isaacs GD, Zhang T, Siepel AC, et al Genomic Analyses of Transcription Factor Binding, Histone Acetylation, and Gene Expression Reveal Mechanistically Distinct Classes of Estrogen-Regulated Promoters. Mol Cell Biol. 2007;27(14):5090–104. 10.1128/MCB.00083-07 17515612 PMC1951957

[20] Clayton AL, Hazzalin CA, Mahadevan LC. Enhanced Histone Acetylation and Transcription: A Dynamic Perspective. Cell. 2006;23(3):289–96. 10.1016/j.molcel.2006.06.017 16885019

[21] Swarthout JT, D'Alonzo RC, Selvamurugan N, Partridge NC. Parathyroid hormone-dependent signaling pathways regulating genes in bone cells. Gene. 2002;282(1–2):1–17. 11814673 10.1016/s0378-1119(01)00798-3

[22] Doggett TA, Swarthout JT, Jefcoat SC Jr., Wilhelm D, Dieckmann A, Angel P, et al Parathyroid Hormone Inhibits c-Jun N-Terminal Kinase Activity in Rat Osteoblastic Cells by a Protein Kinase A-Dependent Pathway. Endocrinology. 2002;143(5):1880–8. 10.1210/endo.143.5.8759 11956171

[23] Suttamanatwong S, Franceschi RT, Carlson AE, Gopalakrishnan R. Regulation of matrix Gla protein by parathyroid hormone in MC3T3‐E1 osteoblast‐like cells involves protein kinase A and extracellular signal‐regulated kinase pathways. Journal of Cellular Biochemistry. 2007;102(2):496–505. 10.1002/jcb.21314 17407158

[24] Takami M, Cho ES, Lee SY, Kamijo R, Yim M. Phosphodiesterase inhibitors stimulate osteoclast formation via TRANCE/RANKL expression in osteoblasts: possible involvement of ERK and p38 MAPK pathways. FEBS letters. 2005;579(3):832–8. 10.1016/j.febslet.2004.12.066 15670856

[25] Wadhwa S, Choudhary S, Voznesensky M, Epstein M, Raisz L, Pilbeam C. Fluid flow induces COX-2 expression in MC3T3-E1 osteoblasts via a PKA signaling pathway. Biochemical and Biophysical Research Communications. 2002;297(1):46–51. 10.1016/S0006-291X(02)02124-1. 12220506

[26] Matsushita T, Chan YY, Kawanami A, Balmes G, Landreth GE, Murakami S. ERK1 and ERK2 play essential roles in osteoblast differentiation and in supporting osteoclastogenesis. Mol Cell Biol. 2009:MCB.01549-08. 10.1128/mcb.01549-08 19737917 PMC2772724

[27] Datta NS, Kolailat R, Fite A, Pettway G, Abou-Samra AB. Distinct roles for mitogen-activated protein kinase phosphatase-1 (MKP-1) and ERK-MAPK in PTH1R signaling during osteoblast proliferation and differentiation. Cellular Signalling. 2010;22(3):457–66. 10.1016/j.cellsig.2009.10.017 19892016 PMC2795117

[28] Homme M, Schmitt CP, Mehls O, Schaefer F. Mechanisms of Mitogen-Activated Protein Kinase Inhibition by Parathyroid Hormone in Osteoblast-Like Cells. J Am Soc Nephrol. 2004;15(11):2844–50. 10.1097/01.ASN.0000143472.13214.2C 15504937

[29] Hatton JP, Pooran M, Li C-F, Luzzio C, Hughes-Fulford M. A short pulse of mechanical force induces gene expression and growth in MC3T3-E1 osteoblasts via an ERK 1/2 pathway. J Bone Miner Metab. 2013;18(1):58–66. 10.1359/jbmr.2003.18.1.58 12510806

[30] Song L, Zhao J, Zhang X, Li H, Zhou Y. Icariin induces osteoblast proliferation, differentiation and mineralization through estrogen receptor-mediated ERK and JNK signal activation. European Journal of Pharmacology. 2013;714(1):15–22. 10.1016/j.ejphar.2013.05.039. 23764463

[31] Luo X-H, Guo L-J, Yuan L-Q, Xie H, Zhou H-D, Wu X-P, et al Adiponectin stimulates human osteoblasts proliferation and differentiation via the MAPK signaling pathway. Experimental Cell Research. 2005;309(1):99–109. 10.1016/j.yexcr.2005.05.021. 15963981

[32] Ljii H, Ung CY, Ma XH, Li BW, Low BC, Cao ZW, et al Simulation of crosstalk between small GTPase RhoA and EGFR-ERK signaling pathway via MEKK1. Bioinformatics. 2009;25(3):358–64. 10.1093/bioinformatics/btn635 19074159

[33] Gesty-Palmer D, Chen M, Reiter E, Ahn S, Nelson CD, Wang S, et al Distinct beta-Arrestin- and G Protein-dependent Pathways for Parathyroid Hormone Receptor-stimulated ERK1/2 Activation. J Biol Chem. 2006;281(16):10856–64. 10.1074/jbc.M513380200 16492667

[34] Marinissen MJ, Gutkind JS. Regulation of gene expression by the small GTPase Rho through the ERK6 (p38γ) MAP kinase pathway. Genes & development. 2001;15:535–53. 11238375 10.1101/gad.855801 PMC312639

[35] Hamamura K, Swarnkar G, Tanjung N, Cho E, Li J, Na S, et al RhoA-Mediated Signaling in Mechanotransduction of Osteoblasts. Connective Tissue Research. 2012;53(5):398–406. 10.3109/03008207.2012.671398 22420753

[36] Singh ATK, Gilchrist A, Voyno-Yasenetskaya T, Radeff-Huang JM, Stern PH. Galpha12/Galpha13 subunits of heterotrimeric G proteins mediate PTH activation of PLD in UMR-106 osteoblastic cells. Endocrinology. 2005;146(5):2171–5. 10.1210/en.2004-1283 15705779

[37] Duncan R, Turner C. Mechanotransduction and the functional response of bone to mechanical strain. Calcified tissue international. 1995;57(5):344–58. 8564797 10.1007/BF00302070

[38] Zhang J, Ryder KD, Bethel JA, Ramirez R, Duncan RL. PTH‐Induced Actin Depolymerization Increases Mechanosensitive Channel Activity to Enhance Mechanically Stimulated 2+ Signaling in Osteoblasts. Journal of Bone and Mineral Research. 2006;21(11):1729–37. 10.1359/jbmr.060722 17002579

[39] Egan JJ, Gronowicz G, Rodan GA. Parathyroid hormone promotes the disassembly of cytoskeletal actin and myosin in cultured osteoblastic cells: mediation by cyclic AMP. Journal of cellular biochemistry. 2004;45(1):101–11. 10.1002/jcb.240450117 1848561

[40] de Leseleuc L, Denis F. Inhibition of apoptosis by Nur77 through NF-[kappa]B activity modulation. Cell Death Differ. 2005;13(2):293–300. 10.1038/sj.cdd.4401737 16082387

[41] Lammi J, Aarnisalo P. FGF-8 stimulates the expression of NR4A orphan nuclear receptors in osteoblasts. Molecular and Cellular Endocrinology. 2008;295(1–2):87–93. 10.1016/j.mce.2008.08.023 18809462

[42] Zhang X, Schwarz EM, Young DA, Puzas JE, Rosier RN, O'Keefe RJ. Cyclooxygenase-2 regulates mesenchymal cell differentiation into the osteoblast lineage and is critically involved in bone repair. The journal of clinical investigation. 2002;109(11):1405–15. 10.1172/JCI15681 12045254 PMC151001

[43] Le Goff B, Blanchard F, Berthelot J-M, Heymann D, Maugars Y. Role for interleukin-6 in structural joint damage and systemic bone loss in rheumatoid arthritis. Joint Bone Spine. 2010;77(3):201–5. 10.1016/j.jbspin.2010.03.002. 20444632

[44] Palmqvist P, Persson E, Conaway HH, Lerner UH. IL-6, Leukemia Inhibitory Factor, and Oncostatin M Stimulate Bone Resorption and Regulate the Expression of Receptor Activator of NF-{kappa}B Ligand, Osteoprotegerin, and Receptor Activator of NF-{kappa}B in Mouse Calvariae. J Immunol. 2002;169(6):3353–62. 12218157 10.4049/jimmunol.169.6.3353

[45] Ishimi Y, Miyaura C, Jin CH, Akatsu T, Abe E, Nakamura Y, et al IL-6 is produced by osteoblasts and induces bone resorption. The Journal of Immunology. 1990;145(10):3297–303. 2121824

[46] Theoleyre S, Wittrant Y, Tat SK, Fortun Y, Redini F, Heymann D. The molecular triad OPG/RANK/RANKL: involvement in the orchestration of pathophysiological bone remodeling. Cytokine & growth factor reviews. 2004;15(6):457–75. S1359-6101(04)00074-7. 15561602 10.1016/j.cytogfr.2004.06.004

[47] Tullai JW, Schaffer ME, Mullenbrock S, Sholder G, Kasif S, Cooper GM. Immediate-Early and Delayed Primary Response Genes Are Distinct in Function and Genomic Architecture. Journal of Biological Chemistry. 2007;282(33):23981–95. 10.1074/jbc.M702044200 17575275 PMC2039722

[48] Huang C, Xue M, Chen H, Jiao J, Herschman HR, O'Keefe RJ, et al The Spatiotemporal Role of COX-2 in Osteogenic and Chondrogenic Differentiation of Periosteum-Derived Mesenchymal Progenitors in Fracture Repair. PLOS ONE. 2014;9(7):e100079 10.1371/journal.pone.0100079 24988184 PMC4079554

[49] Tokushima T, Sato T, Morita I, Murota S. Involvement of prostaglandin endoperoxide H synthase-2 in osteoclast formation induced by parathyroid hormone. Adv Exp Med Biol. 1997;433:307–9. 9561158 10.1007/978-1-4899-1810-9_65

[50] Hou G, Guo C, Song G, Fang N, Fan W, Chen X, et al Lipopolysaccharide (LPS) promotes osteoclast differentiation and activation by enhancing the MAPK pathway and COX-2 expression in RAW264.7 cells. International Journal of Molecular Medicine. 2013;32(2):503–10. 10.3892/ijmm.2013.1406 23740407

[51] Marshall CJ. Specificity of receptor tyrosine kinase signaling: Transient versus sustained extracellular signal-regulated kinase activation. Cell. 1995;80(2):179–85. 10.1016/0092-8674(95)90401-8. 7834738

[52] Shenoy SK, Lefkowitz RJ. Angiotensin II–Stimulated Signaling Through G Proteins and β-Arrestin. Science's STKE. 2005;2005(311):cm14–cm. 10.1126/stke.3112005cm14 16304060

[53] Marshall CJ. Specificity of receptor tyrosine kinase signaling: Transient versus sustained extracellular signal-regulated kinase activation. Cell. 80(2):179–85. 10.1016/0092-8674(95)90401-8 7834738

[54] Sneddon WB, Friedman PA. {beta}-Arrestin-Dependent Parathyroid Hormone-Stimulated Extracellular Signal-Regulated Kinase Activation and Parathyroid Hormone Type 1 Receptor Internalization. Endocrinology. 2007;148(8):4073–9. 10.1210/en.2007-0343 17525124

[55] Radeff JM, Nagy Z, Stern PH. Rho and Rho Kinase Are Involved in Parathyroid Hormone-Stimulated Protein Kinase C alpha Translocation and IL-6 Promoter Activity in Osteoblastic Cells. Journal of Bone and Mineral Research. 2004;19(11):1882–91. 10.1359/JBMR.040806 . 15476589

[56] Mehrotra M, Saegusa M, Wadhwa S, Voznesensky O, Peterson D, Pilbeam C. Fluid flow induces Rankl expression in primary murine calvarial osteoblasts. Journal of Cellular Biochemistry. 2006;98(5):1271–83. 10.1002/jcb.20864 16514640

