# Supplementary figures and images for: Different duration of parathyroid hormone exposure distinctively regulates primary response genes Nurr1 and RANKL in osteoblasts
Source: PLoS One. 2018 Dec 21;13(12):e0208514. doi: 10.1371/journal.pone.0208514 (PMC6303058; doi:10.1371/journal.pone.0208514)

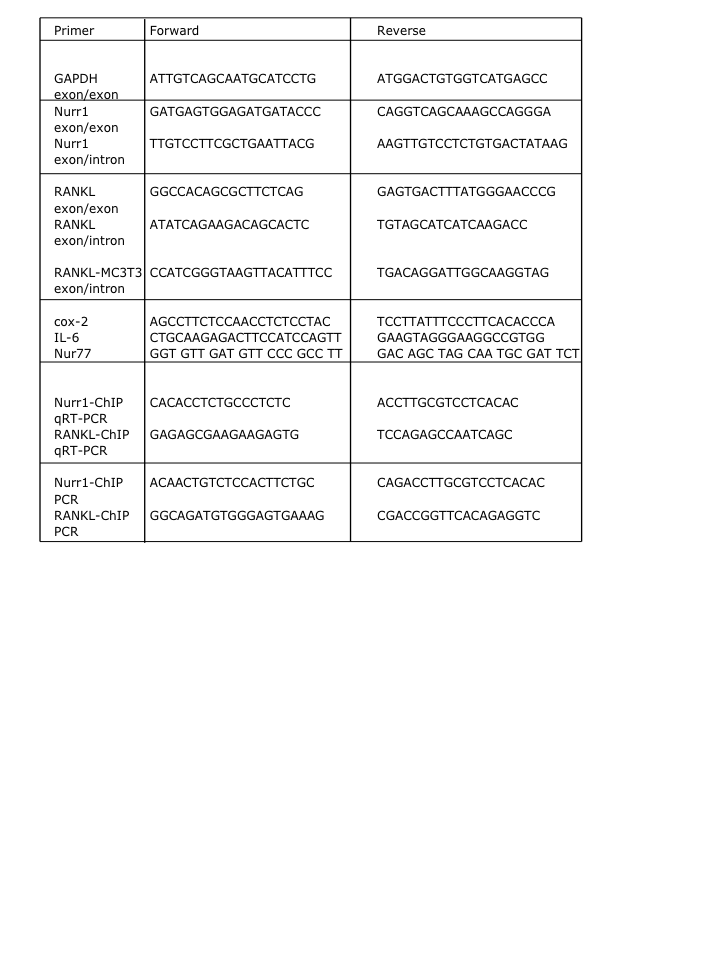

Supplement: S1 Table — (TIFF) [file pone.0208514.s001.tiff]

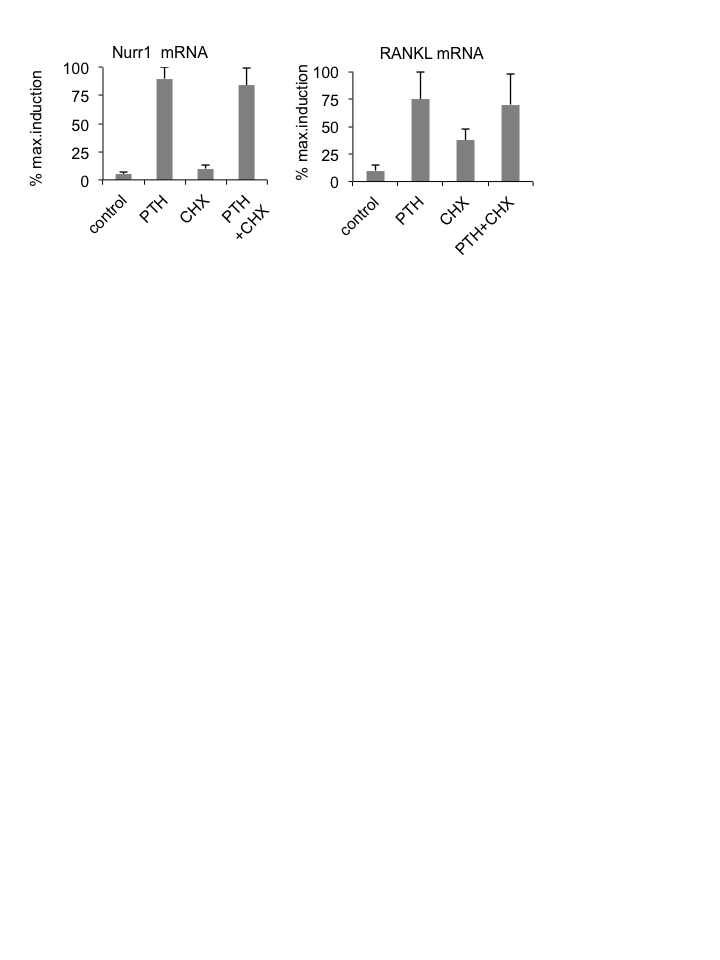

Supplement: S1 Fig — qPCR analysis of Nurr1 (left) and RANKL (right) mRNA expression in MC3T3-E1 cells. Cells were pretreated with 3μg/ml cycloheximide for 0.5 hour and treated with 10 nM PTH treatment for 2 hours. Results indicated that Nurr1 and RANKL are PTH-induced primary response genes in MC3T3-E1 cells (n = 5, mean±SEM, *p<0.05, **p<0.01). (TIFF) [file pone.0208514.s002.tiff]

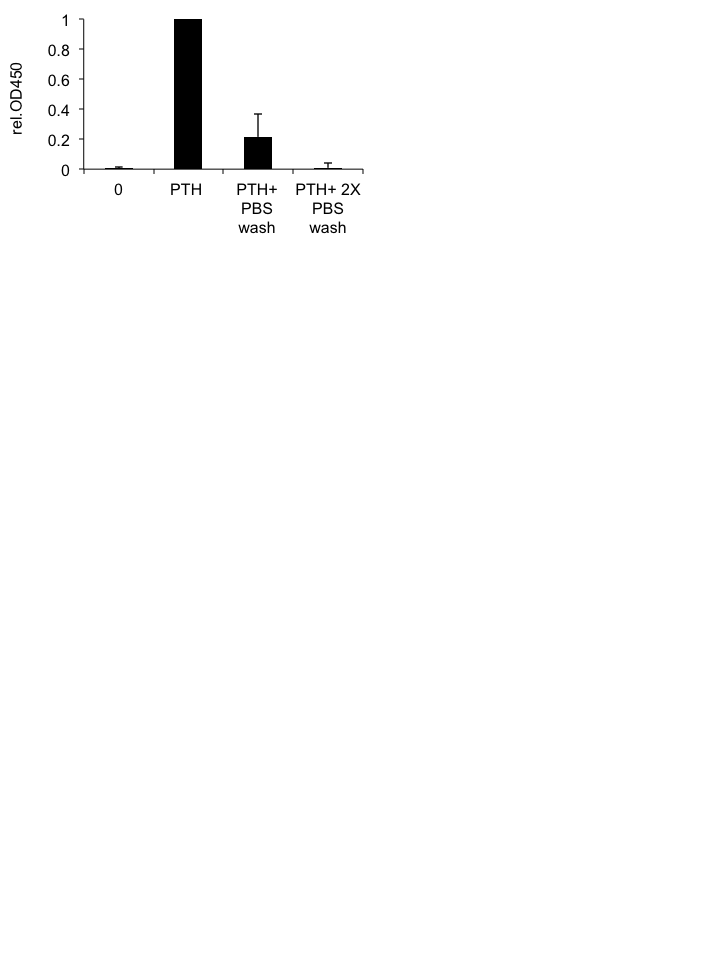

Supplement: S2 Fig — pOBs were treated with PTH for 1 hour followed by indicated number of PBS washes and subjected to PTH ELISA assay. Assay was repeated three times to show. (TIFF) [file pone.0208514.s003.tiff]

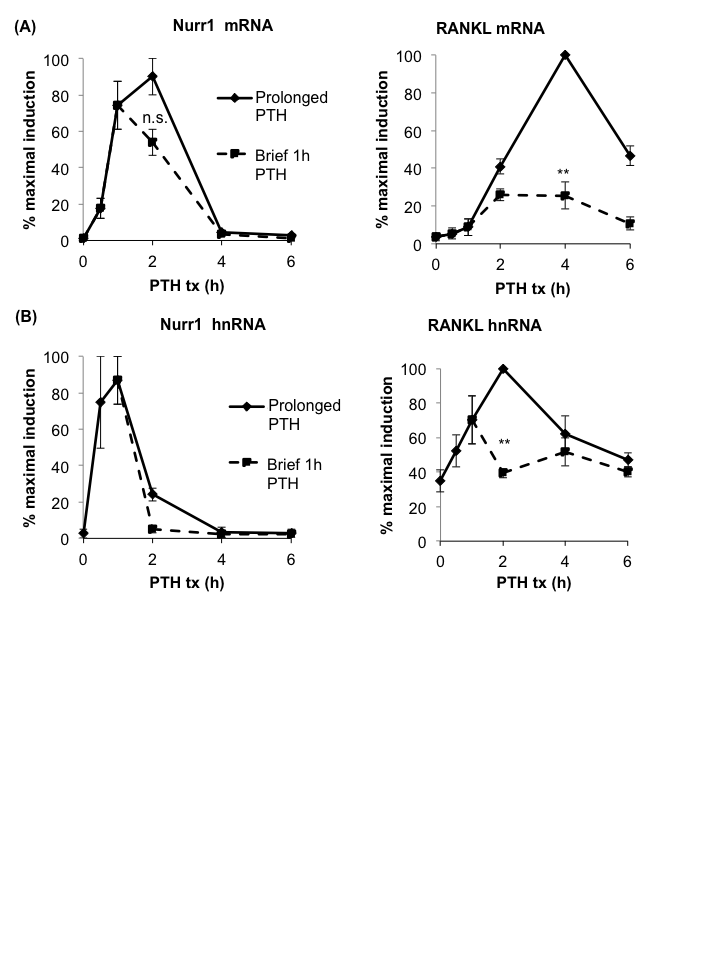

Supplement: S3 Fig — (A,B) qPCR analysis of Nurr1 (left) and RANKL (right) mRNA (A) and hnRNA level (B) in MC3T3-E1 treated with brief or prolonged PTH. MC3T3-E1 cells were treated with 10 nM PTH for 1 hour in 6-hour time course, washed twice with PBS, either changed into PTH-free medium for brief PTH treatment or PTH-containing medium for prolonged PTH, then prepared for qPCR (n = 3, mean±SEM, **p<0.01). (TIFF) [file pone.0208514.s004.tiff]

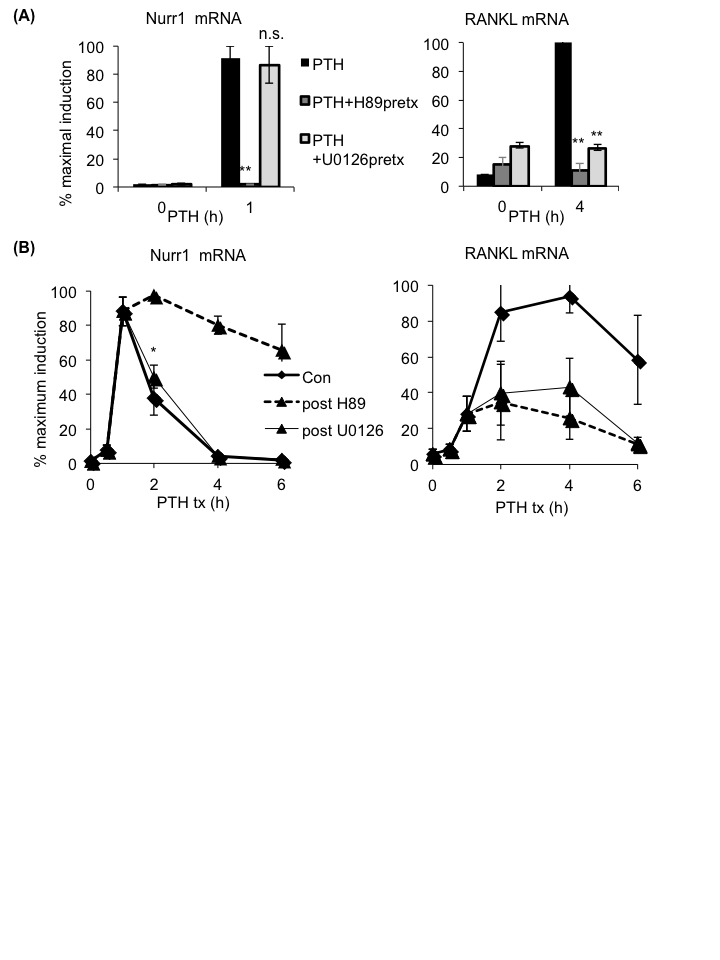

Supplement: S4 Fig — (A,B) qPCR analysis of PTH-induced Nurr1 (left) and RANKL (right) mRNA level in MC3T3-E1 cells pre- or post-treated with 30 μM PKA inhibitor H89 or 10μM MEK/ERK inhibitor U0126. Pre-treatment (A) was done 15 minutes prior to PTH, and post-treatment (B) was done 1 hour after PTH treatment for indicated hours (n = 3, mean±SEM, *p<0.05,**p<0.01). (TIFF) [file pone.0208514.s005.tiff]
